# Examining students' current academic motivation in relation to peer interactions and social environment in the classroom using the Experience Sampling Method

**DOI:** 10.1111/bjep.70002

**Published:** 2025-06-14

**Authors:** Margarita Knickenberg, Carmen L. A. Zurbriggen

**Affiliations:** ^1^ Faculty of Arts and Humanities, Institute of Educational Science Paderborn University Paderborn Germany; ^2^ Department of Special Education University of Fribourg Fribourg Switzerland

**Keywords:** current academic motivation, Experience Sampling Method, multilevel analyses, peer interaction, social classroom climate

## Abstract

**Introduction:**

Current academic motivation is affected by personal and situational factors. This highlights the dynamic nature of academic motivation, which is shaped by its social contexts, particularly by peers at school.

**Aims:**

We investigated the relationships between peer interactions and three aspects of students' current academic motivation (positive activation, enjoyment of learning and concentration) in real learning situations in the classroom. We also examined whether and to what extent aspects of the social environment within the class (social classroom climate, the perceptions of peers and teachers as motivators) affected current motivation.

**Sample:**

The study involved *N*_L2_ = 145 fifth graders in secondary schools, who completed a total of *N*_L1_ = 3099 (*M* = 21.4 per student) short questionnaires on tablet computers during class in one school week.

**Method:**

The Experience Sampling Method was used to simultaneously measure students' aspects of current motivation and their peer interactions in class. In addition, the students reported on their social classroom climate and their perceptions of peers and teachers as motivators using a conventional questionnaire. Multilevel structural equation models were specified.

**Results:**

Results revealed considerable variability in aspects of current motivation. Students showed higher levels of current academic motivation when they interacted with peers compared to learning situations in which they did not interact with peers (i.e. when they studied alone), when they perceived a positive social classroom climate and when they perceived their peers as supportive.

**Discussion:**

The study underscores the situational dependence of students' current academic motivation and the central role of peers in aspects of current academic motivation.

## INTRODUCTION

Academic motivation is considered one of the most important prerequisites for learning and thus one of the most central concepts in educational research (Urhahne & Wijnia, 2023). Most research to date agrees that academic motivation is not (only) a stable characteristic of an individual, but rather a current state that varies over the course of a lesson, a school day or a school year, depending on the interplay of various internal (personal) and external (situational) factors (e.g., Kramer et al., 2024; Opdenakker et al., 2012; Witte et al., 2024).

One of these external factors concerns social relationships, particularly those with peers (Martin & Dowson, 2009). The impact of peers on academic motivation is a crucial issue in educational psychology (Kindermann, 2011). Peers play an essential role in the social, cognitive, affective and behavioural development of children and adolescents (Rubin et al., 2015). They facilitate the acquisition of skills necessary for effective interpersonal interactions, including the ability to navigate complex social dynamics, balance individual needs with those of others and seek and utilize assistance and support. Additionally, they provide a context for learning how to navigate a larger peer culture and cope with challenging situations (Wentzel, 2005). Kindermann (2016) emphasizes the immense importance of peers in academic motivation, stating that ‘(nearly) all components [of motivation] can be affected by interaction with peers’ (p. 32). Thus, exploring the complex relationships between aspects of students' peer environment in the classroom and aspects of current motivation represents a major research desideratum in educational psychology. In this study, we aim to investigate how social interactions with peers in everyday classroom situations relate to students' current academic motivation, which we conceptualize as a situational and variable state. Specifically, we consider both observable peer interactions and perceptions of the social classroom environment to gain a more nuanced understanding of how different social dimensions in class are associated with aspects of current motivation. The study of the relationship between peers and academic motivation is often approached from two perspectives (Wentzel & Muenks, 2016): On the one hand, they are considered in the context of peer membership (such as membership in a particular peer group, social status or the nomination of close peers), and on the other hand, they are investigated in the form of structured interactions with peers, such as the study of the effectiveness of cooperative learning settings.

The present study adds to this body of work by focusing on current peer interactions during regular classroom learning activities and by incorporating students' subjective perceptions of the social classroom climate, teacher and peer support as context‐level variables. Our research thus seeks to connect dynamic, situational peer interactions with broader social perceptions, and to relate both to students' motivational states in the classroom. This study thereby extends previous research that has predominantly examined static peer‐group characteristics or aggregated classroom climate measures without considering *in situ* motivational states.

### (Current) Academic motivation in class

Several prominent motivational theories have been developed in educational research, each with a different focus, to describe, explain and predict multiple facets of learning behaviour and academic outcomes (Urhahne & Wijnia, 2023). We situate the research presented in this paper within the following theoretical frameworks: Self‐determination theory (SDT), for example, focuses on people's innate growth tendencies and the fulfilment of their basic psychological needs, which form the basis of their (general) motivation. SDT also underscores the importance of a sense of belonging or relatedness as a basic psychological need, alongside the need for autonomy and the experience of competence (Ryan & Deci, 2000). In this framework, relatedness is defined as the need to feel a sense of belonging and connectedness with others, while autonomy describes the need to have control over one's interactions with the environment. The ability to perform adequately describes the concept of competence. These needs are interrelated in such a way that the fulfilment of one need promotes the fulfilment of the others (Grolnick et al., 1991).

While SDT primarily (but not exclusively) refers to personal factors of the individual to explain academic motivation (Urhahne & Wijnia, 2023), the General Motivation Model (Heckhausen & Rheinberg, 1980) also considers situational factors. According to this theory, an individual's motivation to pursue a particular goal is influenced by personal and situational factors, including the expected outcomes of actions and their consequences. By considering situational factors, intra‐individual differences can be identified and motivational outcomes that might otherwise be generalized can be attributed to specific situations (Heckhausen & Heckhausen, 2018).

The theoretical framework of flow experience (Csikszentmihalyi, 1975), as another fundamental theoretical concept, also emphasizes the variability of academic motivation by considering flow as a state. Experiencing flow in an activity is considered important for the development of academic motivation. In this theory, concentration and enjoyment are seen as key determinants of (current) motivation. According to Csiksentmihalyi and Schiefele (1993), flow describes a heightened and optimal state of mind, characterized by focused concentration and immense enjoyment, in which people lose track of time and surroundings. Flow experiences occur when an individual's challenges and capabilities are perfectly aligned. Building on this foundation, Shernoff and colleagues (Shernoff, 2013; Shernoff et al., 2003) have significantly advanced the application of flow theory in educational settings by using the Experience Sampling Method to examine students' moment‐to‐moment engagement in the classroom. Their findings highlight the relevance of optimal learning environments that foster concentration, enjoyment and perceived challenge, thereby offering empirical support for the connection between classroom conditions and academic motivation.

Based on these theoretical considerations, it can be concluded that, on the one hand, academic motivation is highly sensitive to the social context (Deci & Ryan, 2012), which is strongly determined by peers, especially during adolescence and at school. On the other hand, academic motivation is highly variable and situational (Pekrun & Marsh, 2022) and can therefore also be conceptualized as a current state. The latent state–trait theory (Steyer et al., 1999) proposes that a person's behaviour in a specific situation is a composite of stable traits and situational influences. In other words, a trait has an effect on the average level of a state, but specific situations can also lead to deviations. While each of these theories provides a different lens, together they form a coherent theoretical foundation: SDT informs our understanding of social needs and their fulfilment; the General Motivation Model provides a framework for analysing situational variance in motivation while the theoretical framework of flow experience guides our selection of current motivational indicators. Together, they enable us to examine how students' current academic motivation is affected by both individual experiences and social contextual cues in the classroom. At the same time, understanding students' academic motivation as a current (situational) state allows situational and personal characteristics to be considered simultaneously (Dietrich et al., 2022), thus revealing the underlying dynamic processes of academic motivation (Witte et al., 2024).

The variability and situational dependence of academic motivation is also supported by empirical evidence: The results of a study measuring aspects of student and teacher motivation in five lessons in class revealed a ‘lesson‐specific’ variance, which the authors attributed to situation‐specific causes or results of fluctuating interactions between individuals and contexts (Gaspard & Lauermann, 2021). In their multilevel analyses, Martin et al. (2015, 2020) found that a substantial proportion of the variance in students' academic motivation resided at the between‐student level, but also that considerable within‐day (between‐lesson) variance existed. However, there was less variability between days and weeks, indicating a limited amount of stable variance components at these timeframes, while motivation still fluctuated within days. The authors conclude that each new lesson or activity may serve as a potential source of motivation, shaped by the dynamic interplay of personal and situational factors.

The high variability and situational dependence of (aspects of) academic motivation (e.g., current interest experiences, current achievement motivation or goal orientation) are confirmed in further studies in the academic context and could be partly explained by situational factors such as autonomy support or task‐specific perceptions (e.g., Knogler et al., 2015; Tanaka & Murayama, 2014; Tsai et al., 2008; Witte et al., 2024).

### Peers and students' social environment in class

According to the SDT framework (Ryan & Deci, 2000), students experience greater self‐determination and motivation when they feel a sense of belonging, acceptance, respect and support from their peers (i.e., classmates) and their teachers. Peers support encompasses both academic and emotional dimensions, while teacher support is typically defined by autonomy, structure and involvement (Kiefer et al., 2015). Together, these sources of support play crucial roles in fostering students' motivation, engagement and sense of school belonging (Kiefer et al., 2015). They also represent key components of the social classroom climate, which is characterized by mutual support, positive peer relationships and an appreciation of diversity (Zurbriggen, Hofmann, et al., 2021).

As early as kindergarten age, children increasingly seek support and guidance from their peers (Wentzel & Muenks, 2016). Social support from peers promotes higher levels of engagement and achievement in school (Mikami et al., 2017). They look to their peers' behaviour as a form of normative behaviour and social interaction (Ryan & Shin, 2018). Peers provide children and adolescents with companionship and entertainment, social and emotional support and offer a normative framework for behaviour and a foundation for identity development throughout childhood and adolescence. In essence, peer interaction represents one of the most critical contexts for socialization during adolescence (Csikszentmihalyi et al., 1977).

Empirical evidence also supports the significance of peers for current emotional experiences, which are closely related to students' motivational outcomes: Students are more motivated and engaged in class when working with peers (high positive activation) but also less stressed or nervous (low negative activation) than in individual situations (Knickenberg et al., 2020; Zurbriggen et al., 2018). Positive emotional experiences are in turn reflected in a general sense of well‐being and academic success (Lyubomirsky et al., 2005). Also in primary school and immediately after the transition to secondary schools, a sense of belonging to peers at school is associated with high positive affect (e.g., feeling well; Schmidt et al., 2019, 2020). A recent study on the characteristics of the social environment in direct learning situations showed that students who were currently working in a self‐determined way, who felt a stronger sense of community and who perceived more support from the teacher (understood here as constructive support from the teacher) reported a higher sense of belonging to their peers in class (Ohl & Dumont, 2024).

A 6‐month intervention study with primary school students found that cooperative learning techniques, such as providing peer feedback, led to an increase in their intrinsic academic motivation (Hotea & Turda, 2024). Furthermore, students enjoy social interactions with their peers more when teachers use learner‐centred practices (e.g., involving students in decision making) and encourage students to work together (Luckner & Pianta, 2011; Wentzel & Muenks, 2016). Study results reveal that students who perceive their peers at school as supportive show higher academic engagement and effort (Danielsen et al., 2010; Kiefer et al., 2015; Wentzel et al., 2017). At the same time, a sense of belonging is enhanced when students perceive their teachers and peers as supportive, both cognitively and emotionally (Furrer & Skinner, 2003). Interestingly, findings from one study suggest that while relationships with both peers and teachers are essential for fifth and sixth graders' behavioural engagement (e.g., paying attention in class), teachers, as opposed to peers, are more important in shaping emotional engagement (e.g., enyoment of learning; Kilday & Ryan, 2019).

Academic motivation, operationalized in the Wentzel et al. (2010) study as interest in classroom activities or enjoyment of being in class, is more pronounced when adolescents perceive their teachers and peers as supportive. Teacher support plays an important role in terms of setting high expectations, providing safety, help,and emotional support, while students value high expectations from their peers. However, it is acknowledged that although peers (e.g., in terms of physical contact, peer support, peer belonging) are associated with positive motivational outcomes, not all students experience the same levels of peer support. Research suggests that the extent to which students experience and perceive peer support and positive interactions varies between individuals and classes (Kilday & Ryan, 2022), highlighting the importance of context.

Although the relevance of peers for (general) academic motivation is well established, individuals' perceptions of their social environment in class, such as the social classroom climate, have (still) received little attention in current research. There is little evidence of a differential link between a more prosocial classroom climate and an increase in individual students' social skills (Hoglund & Leadbeater, 2004). Research suggests that fostering academic motivation through a positive social classroom climate can be highly successful, as children in a more prosocial context are more likely to receive peer support, cooperative forms of learning play a greater role, and there is generally more empathy and compassion (e.g., Wang et al., 2020). In this respect, there is a consensus in research that the social classroom climate is decisively determined not only by teachers, but also, and especially, by peers (e.g., Patrick et al., 2007).

### The current study

As outlined in the theoretical and empirical background, associations between peer interactions, aspects of the social environment and academic motivation can be demonstrated. However, further research is needed, particularly considering the variability of motivation. Furthermore, the question of which aspects of students' motivational experiences are affected by peers remains unanswered (Wentzel et al., 2017). For this reason, the aim of the present study was to examine the effects of peers and students' social environment on aspects of their current academic motivation in class. To this end, a pilot study was conducted using the Experience Sampling Method (ESM; e.g., Hektner et al., 2007), which allows for situational assessment of motivation and accounts for variability in current motivation.

Specifically, we addressed the following research questions:

To what extent do peer interactions in the classroom explain students' current motivation (RQ1)?

To what extent do relatively stable aspects of the classroom's social environment—such as the social classroom climate and students' perceptions of peers and teachers as motivators—affect students' current academic motivation (RQ2)?

## METHODS

### Sample and procedure

The pilot study involved *N*_L2_ = 145 fifth graders (*M* = 10.97 years, *SD* = .09; 56.7% male) from six classes in two secondary schools^1^ in the German federal state of North Rhine‐Westphalia. Data collection took place in the second term of the school year after entering secondary school. Written informed consent was obtained from the students and their primary caregivers.

Two approaches were used to collect data from the students: To meet the understanding of academic motivation as a current state, the Experience Sampling Method (ESM; Hektner et al., 2007) was applied. ESM belongs to the intensive longitudinal methods (Walls & Schafer, 2006) and is an approach to assess and describe the current states of individuals. ESM captures subjective experiences such as current motivation in real situations with as little measurement bias as possible. The real‐time and *in situ* measurement reduces retrospective effects (Zurbriggen, Jendryczko, et al., 2021), while capturing situational and individual characteristics (Bolger & Laurenceau, 2013). The experience sampling survey took place in class and lasted one school week (Monday to Friday). Before starting the survey, trained test administrators explained the main idea and procedure of the ESM to the students and introduced them to the tablet computers and the application used for data collection. Each student was assigned to a tablet computer (via anonymized codes). The tablet computers were prepared with the offline application ‘movisensXS’ (https://xs.movisens.com/). The application was programmed to send an ESM questionnaire and to trigger an acoustic signal at five random times during lessons spread throughout the school day (for all students in the class at the same time). The ESM data collection was as randomized as possible. For this purpose, the participating classes provided us with their timetable, which we used to narrow down the time period of the signals to the school day (excluding breaks and physical education). In order not to disrupt classes too much, there was a minimum of 45 min (one class period) between each measurement. When the signal sounded, the participants were asked to complete a short questionnaire in the application (approximately 2–4 minutes). The questionnaire was identical for all measurement occasions. Out of a total of 3625 possible short questionnaires (145 students * 25 signals in the school week), a total of *N*_L1_ = 3099 (*M* = 21.4 per student) were completed, which corresponds to a response rate of 85.5%. In addition, the participants completed a conventional questionnaire at the end of the survey.

### Measures

#### Experience sampling measures

##### Aspects of current motivation

As an aspect of current motivation, *positive activation* from the PANAVA short scales (PANAVA‐KS; Schallberger, 2005) was used within the experience sampling survey. The items were developed and validated specifically for the ESM context, are short and easy to understand, and have been used successfully in studies with children and adolescents (Zurbriggen et al., 2018). Positive activation was measured with four items on a 7‐point bipolar Likert scale ranging between opposing pairs of adjectives (e.g., ‘listless vs. highly motivated’). The items were introduced to the students in the ESM questionnaire by asking ‘How did you feel just before the signal sounded?’

Two other aspects of students' current academic motivation, *enjoyment of learning* (three items, e.g., ‘It gives me great pleasure’) and *concentration* (two items, e.g., ‘I am completely absorbed in the matter’) were measured on a 7‐point Likert scale ranging from ‘not at all’ to ‘very much’. The items were developed based on the flow concept (Csikszentmihalyi, 1975) and have already been successfully used in an ESM study with a sample of fifth and sixth graders (Zurbriggen & Venetz, 2016).

#### Peer interaction

As part of the experience sampling survey, students were asked to indicate their current form of social activity in class. More specifically, they were asked who they were currently working with. They could choose between the following answer categories: ‘alone’, ‘in pairs’, ‘in a group’, ‘with the whole class’, ‘I listen to the teacher’ and ‘I listen to (a) classmate(s)’. Based on this information, the dichotomous variable *peer interaction* was created for the analyses: ‘Alone’ means that the student worked individually or listened to a teacher or classmate, whereas the category ‘with peers’ indicates a social interaction with one or more peers in the classroom.

#### Trait measures

##### Aspects of the social environment

As part of the conventional questionnaire, students were asked to rate the *social classroom climate* in their class using a subscale of a standardized questionnaire assessing emotional and social experiences at school (FEESS 3–4; Rauer & Schuck, 2003). The subscale consists of four selected items (e.g., ‘We all stick together in class’; Rauer & Schuck, 2003) based on a 4‐point Likert scale (‘not at all true’ to ‘definitely true’).

In addition, students were asked to indicate the extent to which they perceived their peers and teachers as sources of academic motivation using two subscales of the Relationship and Motivation scale (REMO; Raufelder et al., 2013). The subscales relate to *peers as positive motivators* (e.g., ‘My classmates and I motivate each other to make an effort at school’) and *teachers as positive motivators* (e.g., ‘I will make more of an effort in a subject when I think the teacher believes in me’). For the *peers as positive motivators* scale, the six items (out of nine) with the highest factor loadings in the validation study (Raufelder et al., 2013) were selected for the present study in order to keep the questionnaire as short as possible. The items were introduced with the following question: ‘We would like to know how things are going in your class. Please think about how the following statements apply to you. By classmates we mean all the boys and girls in your class’. The *teachers as motivators* scale remained unchanged with six items introduced by ‘Please think about your teachers in general. How much do you agree with the following statements?’ Responses to both subscales were measured on a 4‐point Likert scale ranging from ‘strongly disagree’ to ‘strongly agree’.

### Statistical procedure

**FIGURE 1:**
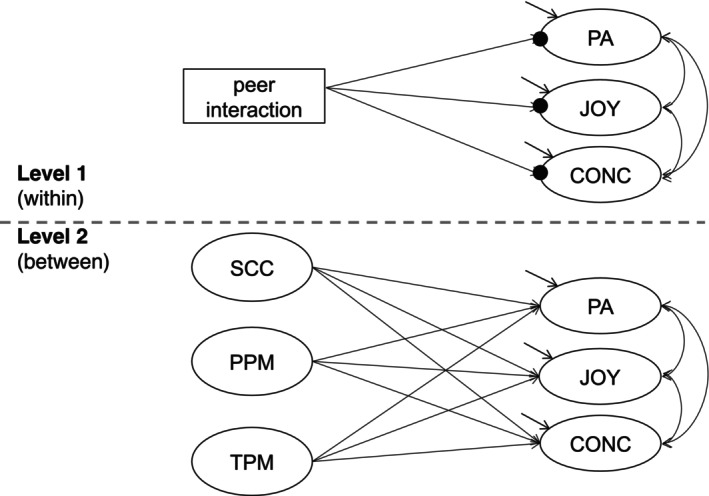
Hypothesized model for explaining variance in students' aspects of motivation at level 1 (within) and level 2 (between). Peer interaction: Dichotomous variable coded ‘alone’ vs. ‘with peers’, PA, Positive activation; JOY, Enjoyment of learning; CONC, Concentration; SCC, Social classroom climate; PPM, peers as positive motivators; TPM, teachers as positive motivators.

Due to the nested data structure (i.e., repeated measures nested within students), multilevel structural equation models (MSEM) with two levels were specified in M*plus* version 8.10 (Muthén & Muthén, 1998–2023). Data from *N*_L1_ = 3099 measurement occasions were included at level 1 (within level). At level 2 (between level), a total of *N*_L2_ = 145 students were considered. At L1, *positive activation, enjoyment of learning* and *concentration* were modelled as dependent latent variables (indicated by ellipses in Figure 1) in a random intercepts model (filled circles represent random intercepts), whereas *peer interaction* was specified as a manifest independent variable (represented by the rectangle). In a consecutive intercepts‐as‐outcomes model (Figure 1) in which the aspects of current motivation were latently aggregated to L2, s*ocial classroom climate, peers* and *teachers as positive motivators* were included as latent independent predictors (between‐level).

The robust maximum‐likelihood estimator (MLR) was used to correct the standard errors and fit indices for non‐normality. Model fit was assessed using the most commonly accepted descriptive fit indices and their cut‐off values. We report the comparative fit index (CFI), the Tucker–Lewis index (TLI), the root mean square error of approximation (RMSEA) and the standardized root mean square residual (SRMR) (Kline, 2005). CFI and TLI values above .90 indicate a reasonable model fit, and values above .95 indicate a good model fit (Hu & Bentler, 1999). For RMSEA, values below .05 are considered a good fit, whereas values between .05 and .08 indicate an adequate fit (Browne & Cudeck, 1993). For SRMR, Hu and Bentler (1999) suggest that values below .08 are considered a good model fit.

## RESULTS

### Descriptive statistics

The unstandardized factor loadings of the manifest items on the latent variables, their intercepts and residual variances are presented in Table 1. The intra‐class correlation coefficients (ICCs; also shown in Table 1) for the *in situ* measures range from .26 to .41, indicating that between 25.8% and 41.0% of the total variance in the dependent variables (positive activation, enjoyment of learning and concentration) can be accounted for by differences between students. This means that there is considerable variance at L2, which underlines the use of a multilevel analytical approach with L2 predictors. At the same time, the ICC values illustrate that a considerable amount of variance can be attributed to factors within the students. As there was little variance at class level in the *in situ* measures with ICCs between .02 and .06, no third level was modelled (e.g., Parrisius et al., 2021; Patall et al., 2016).

**TABLE 1:** Unstandardized factor loading, intercepts, residual variances and reliabilities for all variables in the complete model and intra‐class correlation coefficients (ICC) for the L1 (*in situ*) variables.

Latent variable	Item	Unstandardized factor loading	Intercept	Residual variance	ICC	McDonald's omega (*ω*)	*M* (*SD*)
L1 variables (within level)							
PA^a^	pa1	1.00	5.16***	2.30***	.28	.67^c^	4.68 (1.60)
	pa2	.96***	4.41***	2.55***	.39		
	pa3	1.33***	4.56***	1.67***	.38		
	pa4	1.01***	4.58***	2.61***	.31		
JOY^a^	joy1	1.00	4.36***	1.76***	.32	.69	4.36 (1.60)
	joy2	0.92***	4.17***	2.26***	.33		
	joy3	1.29***	4.56***	1.22***	.41		
CONC^a^	conc1	1.00	4.49***	1.93***	.29	.61	4.43 (1.76)
	conc2	1.36**	4.37***	1.25*	.26		
L2 variables (between level)							
PA (b)^a^	pa1	.89*	5.13***	.43***	.06	.88	
	pa2	1.07*	4.41***	.93***	.03		
	pa3	1.09*	4.53***	.68*	.03		
	pa4	1.09*	4.54***	.41***	.05		
JOY(b)^a^	joy1	.89*	4.31***	.59***	.02	.88	
	joy2	.98*	4.10***	.71***	.03		
	joy3	1.43*	4.50***	.18	.05		
CONC(b)^a^	conc1	.98**	4.48***	.19	.02	.95	
	conc2	1.00**	4.37***	.02	.02		
SCC^b^	scc1	1.00	2.60***	.56***		.71	2.76 (.82)
	scc2	1.02***	2.65***	.58***			
	scc3	1.16***	2.74***	.32***			
	scc4	.74***	2.92***	.52***			
PPM^b^	ppm1	1.00	2.65***	.72***		.85	2.76 (.82)
	ppm2	.98***	2.79***	.61***			
	ppm3	1.22***	2.80***	.44***			
	ppm4	1.07***	2.98***	.51***			
	ppm5	1.08***	2.60***	.65***			
	ppm6	1.14***	2.61***	.65***			
TPM^b^	tpm1	1.00	3.25***	.53***		.80	3.08 (.67)
	tpm2	.96***	3.15***	.55***			
	tpm3	1.06***	3.30***	.33***			
	tpm4	1.40***	2.98***	.49***			
	tpm5	1.21***	2.96***	.48***			
	tpm6	1.25***	2.82***	.66***			

*Note*: *N*_L1_ = 3099; *N*_L2_ = 145. Note that the first factor loading parameter was set to 1 for each latent variable for model identification.

Abbreviations: CONC, concentration; ICC, intraclass correlation coefficient; JOY, enjoyment of learning; PA, positive activation; PPM, peers as positive motivators; SCC, social classroom climate; TPM, teachers as positive motivators.

^a^ Response scale ranging between 1 and 7.

^b^ Response scale ranging between 1 and 4.

^c^ Due to the principle of parsimony and in order to minimize the burden on participants in ESM research, variables are often assessed with single‐item measures or with short scales with few items. This may have implications for the reliability of variables at the L1 level, which is a well‐known and much debated issue in ESM research. The reliability reported here should be considered in relation to the reported ICC.

* *p* ≤ .05;

** *p* ≤. 01;

*** *p* ≤ .001.

First descriptive results show that the students worked alone on more than half of the measurement occasions (55.9%), in pairs on 13.3% of the measurement occasions, in a group or with the class on 20.6% of the measurement occasions and listened to their teacher or classmates on 10.1% of the occasions. The distribution of peer interactions in the dummy variable created for the following analyses is therefore as follows: Students worked alone (respectively without peers) on 66.1% of the measurement occasions and with peers on 33.9% of the measurement occasions.

### Multilevel structural equation models

The multilevel model without predictors at both levels (M0) shows a good model fit. The model fit indices are presented in Table 2. Within a random intercepts model (M1), only L1 predictors were considered, the intercepts of the dependent variables were allowed to vary (random intercept) and the slopes were fixed to be equal between students (fixed slopes). This model (M1) indicates a good fit to the data. The results of M1 (Table 3) show that peer interactions have a positive effect on both students' positive activation (*b* = .17, *p* < .05) and enjoyment (*b* = .16, *p* < .05). This suggests that students enjoy learning more and are more motivated when working with a peer or in groups than when working alone in class. There is no significant effect of peer interaction on students' concentration (*b* = .04, *p* = .70). The variable peer interaction explains 0.5% of the total variance of positive activation and 0.4% of enjoyment of learning within M1 (Table 3).

**TABLE 2:** Model fit indices for the multilevel structural equation models.

	*χ* ^2^	*df*	RMSEA	CFI	TLI	SRMR	AIC	BIC
M0: Intercepts only model (null model without predictors)	86.44	24	.033	.954	.931	.032 (w) .370 (b)	84,885.24	85,057.72
M1: Random intercepts model (L1 predictor)	103.10	30	.032	.952	.928	.031 (w) .000 (b)	84,821.35	85,010.98
M2: Intercepts‐as‐outcomes model (L1 and L2 predictors)	483.19	290	.017	.937	.925	.026 (w) .078 (b)	85,893.36	86,186.14

Abbreviations: CFI, comparative fit index; RMSEA, root mean square error approximation; SRME, standardized root mean square residual; TLI, Tucker–Lewis index.

**TABLE 3:** Unstandardized regression coefficients and determination coefficients for M1 and M2 for the prediction of aspects of students' current motivation.

Predictor	m1: Random intercepts model						M2: Intercepts‐as‐outcomes model					
	PA		JOY		CONC		PA		JOY		CONC	
	*b*	*p*	*b*	*p*	*b*	*p*	*b*	*p*	*b*	*p*	*b*	*p*
Level 1												
Peer interaction^a^	.17	.034	.16	.032	.04	.695	.08	.110	.05	.373	.01	.872
Level 2												
SCC							.28	.044	.18	.122	.22	.399
PPM							.44	.069	.48	.038	−.10	.879
TPM							.33	.211	.10	.705	.11	.879
*R* ^*2*^	.005		.004		.000		.439		.372		.026	

*Note*: *N*_L1_ = 3.099; *N*_L2_ = 145.

Abbreviations: CONC, concentration; JOY, enjoyment of learning; PA, positive activation; PPM, peers as positive motivators; SCC, social classroom climate; TPM, teachers as positive motivators.

^a^ Dichotomous variable coded ‘alone’ vs. ‘with peers’.

When the L2 predictors (social classroom climate, peers and teachers as positive motivators) were added in an intercepts‐as‐outcome model (M2: with random intercepts and fixed slopes), the significant effects of the L1 predictor peer interaction decreased compared to M1. However, the model shows a reasonable model fit (Table 2). The added person‐level (L2) predictors could explain additional variance in the dependent variables. The social classroom climate has a positive effect on students' positive activation (*b* = .28, *p* < .05). Neither the students' perception of peers as positive motivators (*b* = .44, *p* = .07) nor their perception of teachers as positive motivators (*b* = .33, *p* = .21) showed effects on students' positive activation in class. Furthermore, they enjoyed learning more the higher they perceived their peers as positive motivators (*b* = .48, *p* < .05). However, neither the social classroom climate (*b* = .18, *p* = .12) nor the perception of teachers as positive motivators (*b* = .10, *p* = .71) seemed to affect the students' enjoyment of learning. The results indicate that the variable teacher as positive motivators has no significant predictive value on the three aspects of students' current motivation in class. The variance in students' concentration could not be explained significantly by the L1 and L2 predictors (social classroom climate: *b* = .22, *p* = .40; peers as positive motivators: *b* = −.10, *p* = .88; teachers as positive motivators: *b* = .11, *p* = .88). Taking into account the L2 predictors, additional proportions of the total variance of the dependent variables can be explained: Positive activation accounts for almost 44% of the total variance, enjoyment of learning for 37% and concentration for <3% (Table 3).

## DISCUSSION

The aim of this study was to examine the relationships of social interactions with peers and students' current motivation in class (RQ1). The findings revealed considerable variability in the three aspects of current motivation, namely positive activation, enjoyment of learning and concentration, both within and between students, highlighting the intra‐variability and thus the situational dependency of students' current motivation. Accordingly, social interactions with peers were analysed as a (single) situational characteristic predicting students' motivation. The results tended to indicate that students were full of energy and enthusiastic (i.e., higher positive activation) while interacting with peers and exhibited greater enjoyment of learning, yet did not demonstrate greater concentration than in learning situations in which they did not interact with their peers (i.e., learning alone). These findings, although statistically significant, revealed only small effect sizes, indicating a relatively modest association between peer interactions and aspects of students' current motivation. Nonetheless, they support previous research suggesting that social interactions with peers can be a meaningful source of positive activation in the classroom context (Knickenberg et al., 2020; Zurbriggen et al., 2018). As the effects and total explained variances are rather small, the question remains as to what other possible factors within a situation can explain the variability in students' current motivation.

However, concentration as an aspect of the flow experience does not seem to be related to peer interactions. Thus, it can be assumed that peer interactions in the classroom are more strongly related to emotional engagement (e.g., enthusiasm and enjoyment of learning) than to behavioural engagement (i.e., concentration). The extent to which behavioural engagement is directly addressed by peer interactions and the peer environment remains unclear. In principle, empirical evidence on the positive effects of peer interactions (e.g. in the form of partner and group work) in the classroom suggests that these interactions are beneficial for academic motivation by creating a sense of belonging and support (Johnson & Johnson, 2009; Slavin, 2016). Previous studies support these findings by showing mainly positive correlations between peers and subjective well‐being, implying that the feeling of being accepted and included tends to appeal particularly to students' emotions (Baumeister & Leary, 1995; Wentzel & Watkins, 2002). Nevertheless, further research is needed to uncover connections between peer interactions and different aspects of current motivation, especially taking into account further, situation‐specific characteristics and also further characteristics of the peer interaction itself. Understanding these dynamics could provide valuable insights into the design of effective cooperative learning environments.

The second research question (RQ2) examined the relationship between aspects of students' social environment and their current academic motivation. To this end, the social classroom climate, as well as peers and teachers as positive motivators, were included as three predictors in the multi‐level model at the student level. The results revealed that students reported higher levels of positive activation in class when they perceived a positive social classroom climate. Furthermore, students enjoyed class more when they perceived their peers as supportive. The study underscores the central role of peers for aspects of current academic motivation (e.g., Mikami et al., 2017; Schmidt et al., 2019; Wentzel et al., 2017; Zurbriggen et al., 2018).

Regarding the small and statistically not significant relationship between teachers as positive motivators and students' current motivation, our results remain unclear. Research suggests that teacher support has a significant impact on students' (situational) motivation, with positive teacher–student relationships promoting a sense of belonging and security (Pianta et al., 2012; Roorda et al., 2011). In particular, it is apparent that teachers who are empathetic and supportive can increase their students' intrinsic motivation, leading to greater willingness to learn and improved academic performance (Reeve, 2012). Furthermore, research suggests that high‐quality teacher–student interaction not only increases students' situational engagement (Pöysä et al., 2019) but also strengthens their self‐efficacy expectations, which in turn positively influences their motivation (Martin & Rimm‐Kaufman, 2015). At the same time, Givens Rolland (2012) posited in a meta‐analysis on the effects of teacher support on students' affective and cognitive outcomes encompassing studies with students from sixth to twelfth grade that teachers' warmth and support in the classroom had an impact on students' motivational outcomes from seventh grade onwards, but not on those in sixth grade. The author concluded that for the latter, *negative* aspects of the teacher–student relationship have a greater impact on motivation than *positive* aspects (such as teacher support). It can therefore be assumed that the perception of teachers as positive motivators does not yet play a significant role for the fifth graders (as observed in our study). Roorda et al. (2011) explain this on the one hand by the fact that students generally become less motivated in school as they get older and therefore need more support from teachers. On the other hand, secondary school students, unlike primary school students, are usually taught by different teachers with different teaching styles in their daily school life, and the personal relationship with teachers becomes more important over the years. Although the perceived supportiveness of teachers does not directly affect students' motivation in our study, it is plausible to consider an indirect mechanism at play. Teachers manage social interactions in the classroom, model and reinforce rules, norms and expectations for positive social conduct and coordinate social opportunities (Kilday et al., 2023).

In summary, the variability in students' current academic motivation can be attributed to peer‐related aspects of the social environment, particularly those between students. This study provides some evidence that a generally positive classroom climate and perceptions of supportive peers are associated with higher academic motivation. In the future, it would be worth exploring to what extent the effects of peer interactions are related to the social environment (e.g., are peer interactions perceived differently in more supportive classrooms?) and which situational factors (within students) are related to the variability of current academic motivation.

### Limitations and future perspectives

When interpreting the results, it is important to bear in mind that the data collection took place when the fifth graders in this sample had entered secondary school only about half a year earlier. As a result, they may not yet be very familiar with each other or with their teachers and may therefore have difficulties in making judgments about each other.

Future research could pay more attention to the characteristics of the interaction partner(s). According to Shin and Ryan (2014), social interactions are perceived more positively when the interaction partners are similar, for example, in terms of attitudes, learning behaviour and learning performance, a tendency known as homophiliy (McPherson et al., 2001).

Furthermore, the present study does not provide any information on the persistence and autonomy of choice of peer interactions (self‐determined vs. externally controlled). It can be assumed that students are more motivated when they can freely choose their work partners (Krischler et al., 2023). This is also supported by the evidence that the experience of flow is particularly pronounced when students feel empowered in their autonomy (Patrick et al., 2016), that is, when they can make their own decisions. In addition to extending the ESM survey to more accurately reconstruct social interactions in class, it is also possible to use radio frequency identification badges to objectively measure and record the duration and proximity of social interactions between students (Elmer et al., 2019).

When analysing class‐dependent factors, such as classroom climate, it would be important to consider the class as a third level in the model, alongside the individual and situational levels. Empirical evidence suggests that perceptions of peer support and perceptions of respectful and friendly social interactions are highly class dependent (Kilday & Ryan, 2019; Wentzel & Muenks, 2016). Perceptions of teacher support also vary considerably between classes (Danielsen et al., 2010). To address this limitation, a larger sample size (especially at the individual level) would be beneficial in order to analyse more complex models, for example, with cross‐level interactions. Additionally, future studies with larger samples could incorporate class fixed effects to better account for unobserved group‐level differences and further explore potential class‐related influences on within‐student processes.

The effects shown and the total variance explained at the situational level are significant, but rather small. From a methodological point of view, it should be noted that the peer interaction variable was coded as a dummy variable. It is possible that the dichotomization of the response options resulted in a loss of information that would lead to a higher variance explanation of motivation (Holgersson et al., 2014). Furthermore, in our categorization of peer interaction, situations in which students were listening to a classmate were not considered active peer interactions. While this operationalization was theoretically grounded in the assumption that interaction requires reciprocal engagement (Chi, 2009), it may be questioned whether listening to a peer—especially during collaborative tasks—should be classified as a form of social interaction. We acknowledge that such moments may still involve social presence and affect motivational states (e.g., through co‐attention or emotional contagion).^2^ In future research, the operationalization of peer interaction should differentiate more explicitly between partner and group work and whole class interaction, as some students may feel more motivated when working in pairs, whereas others may feel more motivated in larger groups. Additionally, future studies might consider distinguishing between active and passive forms of social engagement more finely, to better capture the diversity of peer‐related experiences in class.

In the present analysis, peer interaction was modelled as a dichotomous variable at level 1 only. An alternative approach would be to include this variable at level 2 as well, representing the proportion of situations in which students collaborated with others. However, as the primary focus of this study was on situational variations in motivation rather than stable individual differences in peer interaction, we opted for a within‐person modelling approach. Future research could further explore potential between‐person differences in the frequency of peer interaction and their role in academic motivation.

Due to organizational constraints, the predictor variables, which are considered relatively stable, were assessed only after the ESM phase using conventional questionnaires. Conducting this assessment beforehand would have been preferable but was not feasible, as the introduction to the ESM procedure and tablet‐based data collection already required significant attention from the students. Future studies could address this by implementing alternative designs to capture predictor variables earlier. At this point, it can be said that peers and peer‐related aspects of students' social environment contribute to the explanation of differences in current academic motivation among young adolescents. In this context, ESM or other types of intensive longitudinal methods offer great potential for investigating relationships between situational and variable aspects such as students' motivation and contextual factors from the peer‐related environment. Accordingly, further research is needed, especially over a longer period of time (e.g., measurement burst design; Sliwinski, 2008). Such research could examine the characteristics of peer interactions at the situational level, thereby providing a more comprehensive understanding of the causal connections between the perceived significance of peers and motivational outcomes. Consequently, this line of inquiry could yield valuable insights for educational practice by offering guidance on how to structure peer interactions and aspects of the social environment within the classroom to promote motivated learning.

## AUTHOR CONTRIBUTIONS

**Margarita Knickenberg:** Conceptualization; funding acquisition; writing – original draft; investigation; methodology; writing – review and editing; formal analysis. **Carmen L. A. Zurbriggen:** Conceptualization; funding acquisition; writing – review and editing; methodology.

## FUNDING INFORMATION

This pilot study was funded by the Faculty of Education of Bielefeld University, Germany.

## CONFLICT OF INTEREST STATEMENT

The authors do not have a conflict of interest to declare.

## Data Availability

The data that support the findings of this study are available upon request from the corresponding author. The data are not publicly available due to privacy or ethical restrictions.

## Footnotes

^1^
In the German education system, in most federal states the transition from primary to secondary school takes place after the fourth grade (at the age of about 10), i.e. fifth graders already attend a secondary school.

^2^
To address this concern, we conducted robustness checks in which situations involving listening to a classmate were re‐categorized as peer interactions. These additional analyses revealed that the pattern of results remained largely stable (no significant changes in model fit and effect sizes).
